# Serum interleukin 1β and sP-selectin as biomarkers of inflammation and thrombosis, could they be predictors of disease severity in COVID 19 Egyptian patients? (a cross-sectional study)

**DOI:** 10.1186/s12959-022-00428-5

**Published:** 2022-12-16

**Authors:** Sara El-Sayed Abd El-Ghani, Reham Mohammad Raafat Hamed, Ragaey Ahmad Eid, Ahmed Yassin Mohammed Ibrahim, Hoda M. Abdel-Hamid, Walaa Abdelrahman, Raghda Ebaid Ibrahim, Manar Mahmoud Abdel-Aziz, Marwa Salah Mohamed

**Affiliations:** 1 Department of Internal Medicine, Faculty of Medicine, Cairo University, Cairo, Egypt; 2 Department of Medical Microbiology and Immunology, Faculty of Medicine, Cairo University, Cairo, Egypt; 3 Department of Tropical Medicine, Faculty of Medicine, Beni-Suef University, Beni-Suef, Egypt; 4 Department of Critical Care Medicine, Faculty of Medicine, Beni-Suef University, Beni-Suef, Egypt; 5 Department of Chest Diseases, Faculty of Medicine, Cairo University, Cairo, Egypt; 6 Department of Rheumatology, Faculty of Medicine, Cairo University, Cairo, Egypt; 7 Department of Clinical & Chemical Pathology, Faculty of Medicine, Beni Suef University, Beni-Suef, Egypt

**Keywords:** COVID-19 severity, IL-1β, sP-selectin, Thromboembolism

## Abstract

**Background:**

Thromboembolism was a chief cause of mortality in 70% of patients with COVID-19. Our objective was to see if serum interleukins 1 beta (IL-1β) and soluble platelets selectin (sP-selectin) could serve as novel markers of thromboembolism in COVID-19 patients.

**Methods:**

This cross sectional study involved 89 COVID-19 patients who were recruited from 1st of February to 1st of May 2021. Clinical and laboratory data were collected, and chest imaging was performed. The levels of IL-1β and sP-selectin were assessed in all cases through ELISA kits. Comparisons between groups were done using an unpaired t-test in normally distributed quantitative variables. In contrast, a non-parametric Mann-Whitney test was used for non-normally distributed quantitative variables.

**Results:**

Severe COVID-19 infection was associated with higher serum levels of CRP, Ferritin, LDH, D dimer, IL-1β and sP-selectin (*P* <  0.001) with significant correlation between levels of IL-1β and sP-selectin *(r 0.37, P <  0.001),* D-dimer *(r 0.29, P 0.006)* and Ferritin *(r 0.5, p <  0.001).*

Likewise, a positive correlation was also found between levels of sP-selectin, D-dimer and Ferritin *(r 0.52, P <  0.001)* (*r* 0.59, *P <  0.001*)*.* Imaging studies revealed that 9 (10.1%) patients developed venous and 14 (15.7%) developed arterial thrombosis despite receiving anticoagulant therapy. Patients with thrombotic events had significantly higher levels of IL-1β, sP-selectin and LDH serum levels. Meanwhile, there was no statistical significance between CRP, D-dimer or Ferritin levels and the development of thrombotic events.

**Conclusion:**

IL-1β and sP-selectin levels can be promising predictors for severe COVID-19 infection and predictable thrombosis.

## Background

In 2019, severe acute respiratory syndrome coronavirus 2 (SARS-CoV-2), a member of Coronaviridae family, resulted in a distinctive infection characterized by acute severe respiratory syndrome (ARDS) which was declared as coronavirus disease of 2019 (COVID-19) pandemic [1].

SARS-CoV-2 infection is frequently linked to coagulopathy and venous thromboembolism (VTE) which promotes tissue injury and multiorgan dysfunction. Venous thrombosis followed by pulmonary embolism (PE) is commonly seen in severe COVID-19 and is found to be associated with increased severity and mortality rate [2].

Although mild flu-like symptoms were reported in most infected cases, viral replication resulted in a severe disease course in 20% of individuals. The reported mortality rate in hospitalized patients with COVID-19 ranges from 4.3 to 15% [3]. Thromboembolism was addressed as a chief cause of mortality in 70% of affected patients [4].

Pathogenesis of COVID-19 infection involves a complex interplay between coagulopathy and endotheliopathy due to viral infection and replication in the target cells with consequent immune and coagulation systems dysregulation [5], leading to uncontrolled inflammatory responses and eventually resulting in the cytokine storm [6]. The fibrinogen level in COVID-19 patients increases by 2–3 folds due to cytokine storm, causing thrombus formation, particularly in severe cases [7]. Moreover, the cytokine storm can damage both epithelial cells and endothelial cells, vascular leakage and finally result in ARDS and death [8].

Many cytokines have been implicated as possible players in a subset of severe patients, such as interlukin-1beta (IL-1β), IL-6, IL-18 and interferon [9]. In response to IL-6 and IL-1ß, the tissue factor is expressed by monocytes, activating the extrinsic pathway of coagulation cascade and suppressing fibrinolysis through the increased release of plasminogen activator inhibitor-1 and decreased activity of urokinase plasminogen activator [10].

In severe COVID-19 infection, pro-coagulant status is mediated through platelet activation and aggregation [11, 12]. Platelets are activated directly by the SARS-CoV-2 spike protein and indirectly through mediators released secondary to endothelial damage [13].

Soluble platelet selectin (sP-selectin) is stored in platelets’ α-granules and in endothelial cells. It acts as an adhesion receptor that initiates the recruitment of leukocytes to the sites of inflammation, tissue injury and immune control [14]. When the extracellular domain is spliced, it is released into circulation and referred to as soluble P-selectin,which mediates the interaction of stimulated endothelial cells or platelets with the white blood cells on the vascular surface [15]. When combined with its ligand P-selectin glycoprotein ligand-1 (PSGL-1), it triggers platelet release and aggregation and mediates the adhesion of platelets to vascular endothelial cells [16].

In the current work, we aim to correlate abnormalities in inflammatory and coagulation pathways which offers opportunities for identifying biomarkers for severe disease and the occurrence of thromboebolism. Combined with clinical risk factors, this will help promptly identify patients at risk of the severe disease requiring early and targeted intervention.

## Methods

### Study design

This prospective cross-sectional study was carried out on 89 COVID-19 infected patients between the 1st and 30th of April, 2021. They were admitted to two government-authorized hospitals to treat COVID-19 patients (Insurance hospital and Beni-Suef University hospital).

### Inclusion criteria

Adult Egyptian patients > 18 years who tested positive by real-time reverse transcriptase-polymerase chain reaction (RT-PCR) assay of the nasopharyngeal sample were enrolled.

### Both sexes are included for the mild, moderate and severe cases

Exclusion criteria included patients whose age was < 18 years, COVID 19 Patients with negative real-time reverse transcriptase-polymerase chain reaction (RT-PCR) assay of nasopharyngeal sample.

According to the interim guidelines of the World Health Organization (WHO) classification [17], COVID-19 infection was categorized into a mild, moderate or severe infection. Typical symptoms without pneumonia or hypoxia were defined as a mild cases, while moderate or severe cases were diagnosed if there were clinical and radiological signs of pneumonia. In moderate infection, patients had to have a saturation of peripheral oxygen (SpO2) ≥ 90% on room air, while one of the following was required to define the severe cases: respiratory rate > 30 breaths/min; severe respiratory distress; or SpO2 < 90% on room air. The treatment protocol followed by the two centers was according to the report of the national institute of health treatment guidelines [18].

### Data collection

Clinical data were obtained, including patient demographics (age, gender, body mass index (BMI)), comorbid conditions (diabetes mellitus, hypertension, chronic pulmonary disease, chronic kidney disease, chronic liver disease, cardiovascular disease) and entire drug history especially anticoagulation and antiplatelets.

Clinical symptoms were reported by asking patients about the presence of constitutional symptoms (fever, bony aches and fatigue), respiratory symptoms (chest pain, cough and dyspnea), gastrointestinal symptoms (diarrhea and abdominal pain) and neurological symptoms (headache and anosmia). The severity of COVID-19 infection was determined based on initial computed tomography of the chest (CT-chest) results and clinical symptoms.

Laboratory assessment included complete blood picture, liver transaminases, serum urea, creatinine, C-reactive protein (CRP), D-dimer, and ferritin were collected within 24 hours of symptoms onset then on deterioration if occurred with documenting a day of deterioration. IL-1β and sP-selectin were assessed in all cases and were sampled at day of diagnosis before initiation of treatment.

Five ml of blood was withdrawn from patients and control groups under complete aseptic condition and added to ethylenediaminetetraacetic acid (EDTA) tubes. Plasma was separated within 30 minutes of collection by centrifugation at 3000 хg. Samples were stored at − 80 °C until assayed for IL-1β and sP-selectin.

### Measurement of plasma IL-1β and plasma sP-selectin

This was done using human IL-1β enzyme-linked immunosorbent assay (ELISA) Kit for quantitatively detecting human IL-1β, catalog numbers BMS224–2 Pub. No. MAN0016591. Rev. C.0, 32 and human sP-selectin Kit for quantitative detection of human sP-selectin, catalog Numbers BMS219–4. This sandwich kit is for the accurate in vitro quantitative detection of human IL-1β and plasma sP-selectin [19].

### Ethical consideration

Ethical approval was obtained from the ethical committee, Faculty of Medicine, Beni-Suef University. Approval NO. FMBSUREC/07032021. Written informed consents were taken from the participants. The study was performed in accordance with the principles of the Declaration of Helsinki.

### Statistical analysis

Data were coded using the statistical package for the Social Sciences (SPSS) version 26 (IBM Corp., Armonk, NY, USA). Data were summarized using mean, standard deviation, median, minimum and maximum for quantitative variables and frequencies (number of cases) and relative frequencies (percentages) for categorical variables. Comparisons between groups were made using an unpaired t-test in normally distributed quantitative variables, while a non-parametric Mann-Whitney test was u sed for non-normally distributed quantitative variables [20]. The Chi-square (χ2) test was performed to compare categorical data. The exact test was used when the expected frequency was less than 5 [21]. The Spearman correlation coefficient [22] revealed correlation between quantitative variables. *P*-values less than 0.05 were considered statistically significant***.***

## Results

### Demographics and clinical characteristics of the study population

The study population included 89 patients who had non-severe and severe COVID-19 infection. They were 44(49.4%) non-severe and 45(50.6%) severe according to WHO classification. 41.6% were males, and 52% were females, with a mean age of 61 ± 10 years. 55 (61.8%) patients received anticoagulation, according to the report of the national institute of health treatment guidelines. However, 23(41.8%) patients had thrombotic events despite anticoagulation, whereas 9 (10.1%) patients developed venous thrombosis (DVT), and 14 (15.7%) patients developed arterial thrombosis (pulmonary embolism) as documented by bilateral lower limb duplex or CTPA (CT pulmonary angiography). Other clinical characteristics of the study population were summarized in (Table 1).

**Table 1:** Clinical characteristics and laboratory data of the study population

		All patients No. = 89	Non-Severe No. = 45	Severe No. = 44	***P***-value
**Age (mean ± SD)**		61.3 ± 10	54.7 ± 8.3	68 ± 6.5	< 0.001
**Gender (No., %)**	**Male**	37(41.6%)	15(33.3)	22(50)	0.11
	**Female**	52(58.4%)	30(66.7)	22(50)	
**Comorbidities (No., %)**					
Diabetes		30 (33.7%)	2(4.4)	28(63.6)	< 0.001
Hypertension		36(40.4%)	4(8.9)	32(72.7)	< 0.001
Chronic pulmonary disease*		2(2.2%)	1(2.2)	1(2.3)	1
Chronic liver disease		2(2.2%)	2(4.5)	0(0)	0.24
Chronic kidney disease		1(1.1%)	1(2.2)	0(0)	0.49
Ischemic Heart disease		4(4.5%)	3(6.8)	1(2.3)	0.36
Thyroid diseases		3(3.4%)	2(4.5)	1(2.3)	0.61
Cancer		2(2.2%)	2(4.5)	0(0)	0.242
**Thrombotic events (No., %)**	Venous (DVT)	9 (10.1%)	0(0)	9(20.5)	0.001
	Arterial (PE)	14 (15.7%)	1(2.2)	13(29.5)	< 0.001
**Method of diagnosis (No., %)**	Duplex	7(7.9%)	0(0)	7(16)	1
	CTPA	14(15.7)	1(2.2)	13(29.5)	
	Both	2(2.2)	0(0)	2(4.5)	
**Received anticoagulation (No., %)**		55(61.8%)	11(24.4)	44(100)	< 0.001
**Anticoagulation type (No., %)**	LMWH	37 (41.6%)	0(0)	37(84.1%)	< 0.001
	DNOA	18 (20.2%)	11(24.4)	7(15.9%)	
**Laboratory investigations (mean** ± **SD)**					
Hemoglobin (gm/dl)		12.5 ± 1.6	13.8 ± 2.2	16.6 ± 3	< 0.001
Total leucocytic count (/cmm)		6.9 ± 3.1	7.5 ± 3.2	6.2 ± 2.8	0.007
Platelets (/ μL)		252 ± 51	250 ± 66	254.7 ± 29.5	0.66
Lymphocytes %		27.2 ± 10.5	31 ± 11	23 ± 8	0.001
Neutrophil %		57 ± 11.4	60.5 ± 12.5	53.4 ± 9	0.003
AST (U/L)		29.8 ± 12.4	29 ± 4.8	30.5 ± 17	0.05
ALT (U/L)		27.5 ± 9.5	26.6 ± 4.1	28.3 ± 12.8	0.35
CRP (mg/L) (median-IQR)		21(1.5–120)	5(1.5–78)	33(17–120)	< 0.001
D dimer (μg/mL) (median-IQR)		0.6(0.1–2.4)	0.4 (0.1–1.7)	0.8(0.3–2.4)	< 0.001
Ferritin (ng/mL)		278 (11–1498)	85(11–900)	645(178–1498)	< 0.001
LDH (U/L)		336 ± 156	269 ± 57	404 ± 193	< 0.001
IL-1β (pg/ml)		15.2 ± 2.9	13.8 ± 2.1	16.6 ± 2.9	< 0.001
sP- selectin (ng/ml)		15 ± 3.2	13 ± 1.8	17 ± 2.8	< 0.001

*Abbreviations:* * *COPD* patient, *DVT* deep venous thrombosis, *PE* pulmonary embolism, *CTPA* CT pulmonary angiogram, *AST* Aspartate Transaminase, *ALT* Alanine Transaminase, *CRP* C-Reactive Protein, *LDH* Lactate Dehydrogenase, *IL-1β* Interleukin 1 beta, *DNOC* Direct New Oral Anticoagulants, *LMWH* Low Molecular Weight Heparin

### Clinical characteristics of patients with non-severe versus severe COVID-19 infection

The mean age of patients with non-severe COVID-19 infection was 54.7 ± 8.3 years, while in severe was 68 ± 6.5, with statistical significance between the two groups (P = < 0.001).

28(63.6%) patients with diabetes and 32(72.7%) patients with hypertension experienced severe COVID-19 infection (*P* <  0.001). In contrast, no significant association was found with other comorbidities such as chronic pulmonary disease, chronic liver and kidney disease, ischemic heart disease, thyroid disease and cancer (Table 1).

### Characteristics of patients with severe COVID-19 infection in relation to laboratory data

Patients with severe COVID-19 infection had significantly lower levels of total leucocyte count, lymphocyte %, neutrophil % and higher levels of CRP, ferritin, D-dimer, LDH, IL-1β and sP-selectin as demonstrated in (Table 1).

**Fig. 1:**
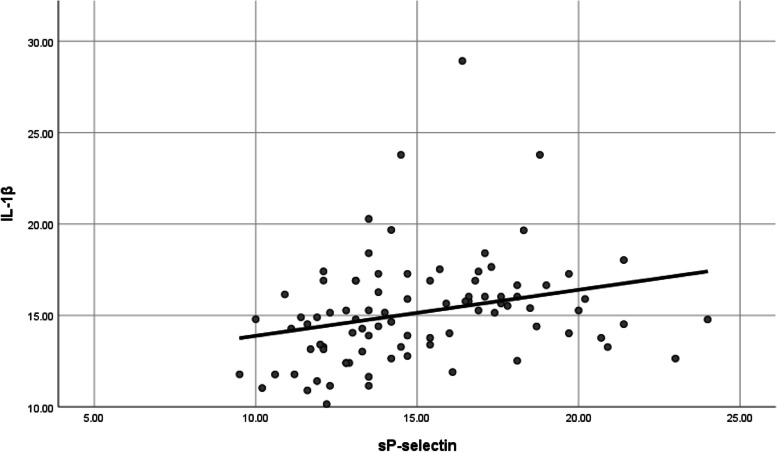
Correlation between IL-1β and sP-selectin in the study population (***r =*** 0.371, *P* value < 0.001)

There was a statistically significant positive correlation between CRP, D-dimer, ferritin, LDH, IL-1β, and sP-selectin serum levels and the severity of COVID-19 infection (Table 2) (Fig. 1).

**Table 2:** Correlation between D dimer, Ferritin, IL-1β and sP-selectin in the study population

		IL-1β	sP-selectin	D dimer
**sP-selectin**	*r*	0.371		
	*P value*	< 0.001		
**D dimer**	*r*	0.292	0.526	
	*P value*	0.006	< 0.001	
**Ferritin**	*r*	0.516	0.594	0.449
	*P value*	< 0.001	< 0.001	< 0.001

### Clinical characteristics of patients without versus with thrombotic events

The mean age of patients without thrombotic events was 64 ± 10, while in patients with thrombotic events was 67 ± 7 years, with no statistically significant difference between the two groups (*P* = 0.18). Also, there was no statistically significant difference between the two groups in terms of gender or preexisting medical comorbidities, as demonstrated in (Table 3).

**Table 3:** Clinical characteristics and laboratory data of patients without and with thrombotic events despite anticoagulation

		All patients No. = 55	Non-Thrombotic No. = 32	Thrombotic No. = 23	***P***-value
**Age (mean ± SD)**		65 ± 9	64 ± 10	67 ± 7	0.18
**Gender (No., %)**	**Male**	26 (47.3%)	17 (53.1%)	9 (39%)	0.305
	**Female**	29(52.7%)	15 (46.9%)	14 (61%)	
**Comorbidities (No., %)**					
Diabetes		28(51%)	13(41%)	15(65%)	0.072
Hypertension		34(62%)	18(56%)	16(70%)	0.316
Chronic pulmonary disease*		2(4%)	2(6%)	0(0)	0.504
Chronic liver disease		2(4%)	2(6%)	0(0)	0.504
Chronic kidney disease		1(2%)	1(3%)	0(0)	1
Ischemic Heart disease		3(5.5%)	1(3%)	2(9%)	0.565
Thyroid diseases		2(4%)	2(6%)	0(0)	0.5
Cancer		2(4%)	1(3%)	1(3%)	1
**Severity of COVID − 19 (No., %)**	**Non-Severe**	11(20%)	9(28%)	2(9%)	0.097
	**Severe**	44(80%)	23(72%)	21(71%)	
**Laboratory investigations (mean** ± **SD)**					
Hemoglobin (gm/dl)		12.2 ± 1.5	12.3 ± 1.6	12 ± 1.3	0.50
Total leucocytic count (/cmm)		6.5 ± 2.8	6.9 ± 2.5	6 ± 3	0.30
Platelets (/ μL)		254 ± 36	247.7 ± 35	263 ± 37	0.11
Lymphocyte %		25 ± 9.4	25.5 ± 9.3	24 ± 9.6	0.52
Neutrophil %		54.4 ± 10	56.9 ± 10.4	50.8 ± 8.6	0.03
AST (U/L)		30.6 ± 15.6	34 ± 19.5	25.7 ± 4.7	0.04
ALT (U/L)		28 ± 12	30.6 ± 14.7	24.6 ± 3.8	0.03
CRP (mg/L)(median-IQR)		32(3–120)	31 (3–59)	32(5–120)	0.33
D dimer (μg/mL)(median-IQR)		0.8(0.3–2.4)	0.7(0.3–1.7)	0.9(0.3–2.4)	0.094
Ferritin (ng/mL)		569(11–1498)	490(11–1300)	655(66–1498)	0.094
LDH (U/L)		381 ± 182	328 ± 130.5	455 ± 218	0.018
IL-1β (pg/ml)		16 ± 3	15 ± 1.8	17.2 ± 3.9	0.028
sP- selectin (ng/ml)		16.6 ± 3	16 ± 3.1	17.7 ± 2.2	0.015

*Abbreviations:* *COPD, *AST* Aspartate Transaminase, *ALT* Alanine Transaminase, *CRP* C-Reactive Protein, *LDH* Lactate Dehydrogenase, *IL-1β* Interleukin 1 beta

### Characteristics of patients with thrombotic events concerning the severity of COVID-19 infection

There was no statistical significance in COVID-19 infection severity and the development of thrombotic events (*P* = 0.09) (Table 3).

### Characteristics of patients with thrombotic events in relation to laboratory data

**Fig. 2:**
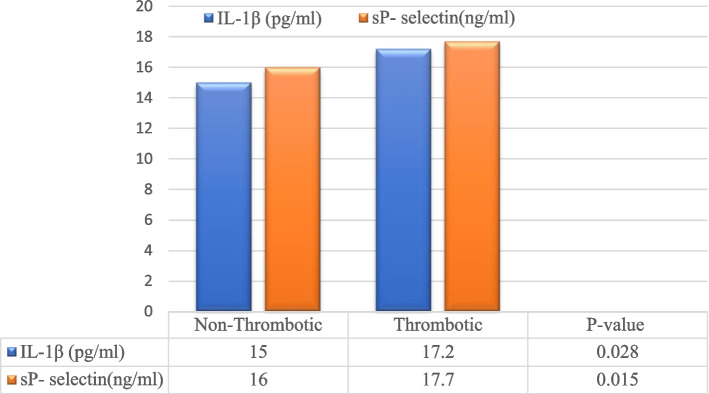
Serum levels of IL-1β and sP-selectin in Non-Thrombotic versus Thrombotic COVID-19 patients

Patients with thrombotic events had significantly higher levels of IL-1β, sP-selectin, and LDH serum levels. Meanwhile, there was no statistical significance between serum levels of CRP, D-dimer or ferritin and the development of thrombotic events (Table 3) (Fig. 2).

## Discussion

This study aims to assess the levels of IL-1β as an inflammatory cytokine and sP-selectin as a novel plasma marker for thromboembolism in COVID-19 patients and correlate abnormalities in inflammatory and coagulation pathways (IL-1β and sP-selectin) to the severity of the disease and the occurrence of venous thromboembolism (VTE) even on anticoagulation therapy. This, combined with clinical risk factors, will help promptly identify patients at risk of the severe disease who require early and targeted intervention.

SARS-CoV-2-Induced endothelial damage releases a variety of soluble markers of endothelial dysfunction, including von Willebrand factor (VWF), soluble E-selectin, P-selectin and thrombomodulin. Such biologically active molecules ultimatepromote platelet activation, which eventually contributes to inflammatory response and thrombotic complications, increasing the severity of COVID-19 [23].

On the assessment of IL-1β and sP-selectin levels and their relation to disease severity, we found a statistically significant increase in their level in the severe group compared to the non-severe group. Also, there is a statistically significant difference in D-dimer, CRP, Ferritin and LDH serum levels between non-severe and severe COVID-19 patients. Huang et al., studied Prothrombin time and D-dimer levels on admission which were higher in ICU patients (median prothrombin time 12·2 s [IQR 11·2–13·4]; median D-dimer level 2·4 mg/L [0·6–14·4]) than non-ICU patients (median prothrombin time 10·7 s [9·8–12·1], *p* = 0·012; median D-dimer level 0·5 mg/L [0·3–0·8], p = 0·0042) [24].

Tang et al. did not report on VTE incidence but noted derangement in coagulation and clotting markers PT, aPTT, D-dimer, fibrin degradation products which were higher in non-survivors [25].

Chiara Agrati et al. investigated sP-selectin plasma concentration as a biomarker of endothelial dysfunction and platelet activation in 46 severe COVID-19 hospitalized patients and found a higher sP-selectin plasma concentration in patients with Covid-19, regardless of intensive care unit (ICU) admission compared to the typical reference values and compared to ten contextually sampled healthy donors [26].

Emre Karsli et al. assessed the relationship between sP-selectin level and the clinical severity of COVID-19 infections in 80 patients, and similar to our study, they divided them into two groups, mild to the moderate group containing 50 patients and the severe group 30 patients, they compared results to 60 non-symptomatic healthy volunteers.

They found that serum sP-Selectin levels in both mild-to-moderate pneumonia and severe pneumonia groups were higher than in the control group, with a statistical significance difference.

They also found that the serum sP-Selectin level was found to be 76.9% sensitive and 51.9% specific to predict the need for intensive care treatment and concluded that sP-selectin can be used as a valuable biomarker in both diagnosing and predicting the need for intensive care treatment of COVID-19 infection [27].

Evolving datasets indicate that cytokines like IL-1β, IL-6, IL-17A, IL-9, transforming growth factor-β (TGF-β) and C-C chemokine ligand 2 (CCL-2) promote thrombosis, and other cytokines such as IL-8, IL-10 and TNF-α help in thrombus resolution [28].

In studying the relationship between the incidence of thrombosis and the levels of IL-1β and sP-selectin, there was a statistically significant correlation between serum levels IL-1β and sP-selectin concerning the occurrence of thrombosis in COVID-19 patients.

Mona M. Watany et al. studied 103 hospitalized COVID-19 patients and 50 healthy volunteer controls. COVID-19 patients were categorized into two groups; group 1, which developed thrombosis during hospitalization and group 2, which did not. Soluble selectins were quantitated using the ELISA technique. Higher levels of sP-selectin, sE-selectin and sL-selectin were detected in COVID-19 patients compared to controls. Significantly, higher levels were found in group 1 compared to group 2 [29].

In addition, we found 21 patients who developed thrombosis among the severe group despite receiving anticoagulation, compared to 2 patients only in the mild group, with a statistically significant difference between both groups, which also intensifies this idea.

So, we suggest evaluating the potential role of IL-1β as a therapeutic target in COVID management. Lorenza Landi et al. studied blockage of interleukin-1β with canakinumab in 88 patients hospitalized for Covid-19 pneumonia. Oxygen-support requirements improved, and overall mortality was 13.6% [30].

## Conclusions

We concluded that IL-1β and sP-selectin levels could be promising predictors for severe COVID-19 infection and predictable thrombosis in the case of higher levels.

We suggest evaluating the potential role of IL-1β as a therapeutic target in COVID management.

Further studies should be directed toward this topic among a more significant number of patients and different ethnic groups. Also case control studies including healthy controls to compare the differences between controls, non severe and severe COVID-19 patients should be performed, which is considered a limitation of our study.

## Data Availability

The datasets used and/or analyzed during the current study are available from the corresponding author on reasonable request.

## Abbreviations

ARDS: Acute severe respiratory distress syndrome
BMI: Body mass index
COVID-19: Coronavirus disease of 2019
CCL-2: C-C chemokine ligand 2
CRP: C-reactive protein
CT-chest: Computed tomography of the chest
CTPA: Computed tomography pulmonary angiography
EDTA: Ethylenediaminetetraacetic acid
ELISA: Enzyme-linked immunosorbent assay
ICU: Intensive care unit
IL-1β: Interlukin-1beta
LDH: Lactate dehydrogenase
PSGL-1: P-selectin glycoprotein ligand-1
PE: Pulmonary embolism
RT-PCR: Real-time reverse transcriptase-polymerase chain reaction
SARS-CoV-2: Severe acute respiratory syndrome coronavirus 2
SpO2: Saturation of peripheral oxygen
sP-selectin: Soluble platelet selectin
SPSS: Statistical package for the Social Sciences
TGF-β: Transforming growth factor-β
TNF: Tumor necrozing factor
VTE: Venous thromboembolism
VWF: Von willebrand factor
WHO: World Health Organization
